# COVID-19 Cases and Deaths among Healthcare Personnel with the Progression of the Pandemic in Korea from March 2020 to February 2022

**DOI:** 10.3390/tropicalmed8060308

**Published:** 2023-06-05

**Authors:** Yeonju Kim, Sung-Chan Yang, Jinhwa Jang, Shin Young Park, Seong Sun Kim, Chansoo Kim, Donghyok Kwon, Sang-Won Lee

**Affiliations:** 1 Division of Public Health Emergency Response Research, Korea Disease Control and Prevention Agency (KDCA), Cheongju 28159, Republic of Korea; yeonju.kim5@gmail.com (Y.K.); npros33@korea.kr (S.-C.Y.); jinhjang@korea.kr (J.J.); young9077@korea.kr (S.Y.P.); sskim0719@korea.kr (S.S.K.); 2 AI/R Lab, AI-Robot Department, University of Science and Technology, Seoul 02792, Republic of Korea; 3 AI/R Lab, Computational Science Center & ASSIST, Korea Institute of Science and Technology, Seoul 02792, Republic of Korea; 4 Division of Epidemiological Investigation Analysis, Korea Disease Control and Prevention Agency (KDCA), Cheongju 28159, Republic of Korea; 5 Public Health Emergency Preparedness, Korea Disease Control and Prevention Agency (KDCA), Cheongju 28159, Republic of Korea; epilsw@korea.kr

**Keywords:** COVID-19, SARS-CoV-2, healthcare personnel, Korea, policy, OECD

## Abstract

Healthcare personnel (HCP) are vulnerable to COVID-19 infection due to their higher risk of contact with infected persons. The numbers of cases and deaths among HCP in Korea were divided into four periods associated with different major variants of SARS-CoV-2: GH clade, Alpha, Delta, and Omicron. To evaluate the implication of HCP infection in Korea, we overviewed the pandemic status in Korea and in other countries: the cases, deaths, excess mortality, and vaccination rates in Germany, Israel, Italy, Japan, the United Kingdom, and the United States. In about two years, there were 10,670 HCP cases among all COVID-19 cases (1.15% of 925,975 cases). HCP cases had a lower death rate (%) compared to that for all cases (0.14 versus 0.75). Nurses were the most infected (55.3%), followed by HCP of other categories (28.8%) and doctors (15.9%), while deaths were mostly reported among doctors (9 out of 15, 60%). Cases among HCP gradually increased, but the death rate decreased as the pandemic progressed. Compared to five of the other countries examined, Korea had a higher incidence of cases but a lower mortality, lower excess mortality, and a higher vaccination rate.

## 1. Introduction

Healthcare personnel (HCP) caring for COVID-19 patients in hospitals or clinics are at the highest risk of SARS-CoV-2 transmission and infection. In particular, in the early period of the pandemic in 2020, when a vaccine was not available and the epidemic characteristics of SARS-CoV-2 were not clearly understood, infection rates among HCP surpassed those among the rest of the population [1,2,3]. The prevalence of SARS-CoV-2 infection was higher in healthcare workers than in blood donors, at 4.04% and 3.04% (RR = 1.33), respectively, in a cohort study in Denmark examining the period from 15 April to 30 April 2020 [2]. In the United States, from 12 February 2020, to 9 April 2020, about 50,000 cases were reported to the CDC; of these, about 9200 patients (19%) were HCP, and 27 HCP died [1]. Among 1097 healthcare workers without an epidemiological link but with mild respiratory symptoms in nine hospitals in the Netherlands between 7 March 2020 and 8 March 2020, laboratory-confirmed cases of COVID-19 accounted for 4.1% (45 cases) among those tested [3]. This prevalence was comparatively higher than that in the general population in the Netherlands—128 cases as of 6 March 2020—and it was suggested that unnoticed transmission in the community and in hospitals was highly possible.

As the COVID-19 pandemic has continued for more than three years, policies regarding COVID-19 management have changed in many countries; for example, non-pharmaceutical interventions have been lifted, and vaccination programs of periodical inoculation are underway. Additionally, the infection rate among HCP might have changed: high infection rates among HCP, including nosocomial infections, have decreased as SARS-CoV-2 variants have evolved to have higher transmission rates and cases have increased in the community. Nevertheless, not many have described the change in infection statistics among HCP throughout the course of the pandemic.

Based on the results of the previous reports, risks of occupational exposure were suggested among HCP, and the necessity of lightening the burden of the disease among HCP was underlined to protect their mental and physical health [4,5,6]. From a qualitative review of 161 published papers regarding socio-ecological experiences among healthcare workers, negative personal emotions were observed in the initial period of the pandemic [5].

In this study, we analyzed the number of COVID-19 cases and deaths among HCP by the progression, with the aim of estimating the magnitude of COVID-19 infection among HCP during the outbreak of two years in Korea. The cases and deaths among HCP were compared with the months characterized by major variants of SARS-CoV-2 over the course. Additionally, deaths over cases due to COVID-19 among HCP and among the whole population were compared.

We also aimed to compare the pandemic profiles in South Korea to those in other countries to facilitate a further understanding of the transmission and management of COVID-19. Therefore, we summarized the number of cases, deaths, and vaccination rates of COVID-19 in selected countries, including Germany, Israel, Italy, Japan, the United Kingdom, the United States, and South Korea. These countries are among those with early reports of COVID-19 cases and which experienced community outbreaks and/or lockdown, as well as introducing vaccination relatively early compared to the global average. In addition, to evaluate systematic factors related to dealing with emerging infectious disease, we reviewed several indicators of social, economic, and public health sectors from published sources [7,8,9,10].

## 2. Materials and Methods

### 2.1. Study Design

For the analysis of HCP, a case series study was designed. In South Korea, COVID-19 diagnosis was based on PCR testing of nasopharyngeal and/or oropharyngeal swap samples in public health centers until 13 March 2022. From 14 March 2022, positive results from rapid antigen tests (RATs) conducted at hospitals and clinics were also considered effective. Information on the demographic and epidemiologic characteristics of all COVID-19 cases was collected mostly by telephone interview by the workers at public health centers nationwide. The patient information was reported through the web-based reporting system for the infectious diseases surveillance system from public health centers to the Korea Disease Control and Prevention Agency (KDCA). Information on the date the test was taken, age, city of residence, sex, whether any symptoms developed, whether the case was in HCP, etc., was collected. Epidemic characteristics were additionally collected through a following epidemiologic investigation, and the variables collected were comorbidity; detailed information of symptoms, including date of onset; route of transmission; and possible contacts. As the number of COVID-19 cases increased steeply in Korea, interview-based reporting and epidemiologic investigation were switched to a self-input system from 7 February 2022. Cases of COVID-19 climbed as each new variant became the major genomic type due to a substantial increase in the transmission rate by 50% to 100% [11], or to lesser neutralization activity to the vaccines [12]. Based on this rationale, we reviewed the variables across each period associated with four dominant variants—GH clade, Alpha, Delta, and Omicron. The genomic surveillance of SARS-CoV-2 by the KCDA was introduced just after the first case of COVID-19 was diagnosed. The coverage of genomic sequencing for the surveillance of SARS-CoV-2 in South Korea was 3.3% in Dec 2020 and increased to 20% from Oct 2021 [13].

The overall infection rate of COVID-19 among HCP in each country was not reported nor comparable in general. Meanwhile, COVID-19 infections among HCP may follow the pandemic status in each country. Therefore, instead, we conducted a narrative review of several variables of COVID-19 in six countries and described the results from two time points (30 June 2021 and 30 June 2022) to examine any changes over time. Several institutions, such as the Johns Hopkins Coronavirus Resource Center, WHO Coronavirus (COVID-19) Dashboard, Institute for Health Metrics and Evaluation (IHME), and Our World In Data, provide information regarding COVID-19 worldwide periodically. Each site has their own distinction based on the objective of data collection and provision. For example, the Johns Hopkins Coronavirus Resource Center provides detailed data limited to the United States, and IHME provides a web-based tool of mathematical modeling. In this study, we downloaded the COVID-19 indicators from OurWorldInData.org, since they include numerous indicators of the COVID-19 pandemic from many countries [7,10]. Data sources used by the OurWorldInData team are data or statistics mainly from international organizations composed by representatives of governments from each government. Their publication has been widely used by researchers, policy makers, journalists, and general people, including students, accounting for 300 million pageviews in 2022. Demographic/socio-economic factors and healthcare profiles were extracted from recent reports from the United Nations [8,9].

### 2.2. Study Population

The inclusion criteria of our analysis were HCP professions in three groups: doctors (medical doctors, dentists, and oriental doctors); nurses (registered nurses and nurse assistants); and others (physical therapists, radiologic technicians, emergency medical technicians, medical laboratory technologists, occupational therapists, psychologists, dental assistants, care workers, pharmacists, herbalists, paramedics, and firefighters), among 925,975 COVID-19 cases in South Korea reported from 21 March 2020 (Sat) to 4 February 2022 (Fri), covering a period of 98 weeks (22 months and 15 days). The exclusion criteria were confirmed cases between 20 January 2020 and 20 March 2020, since the cases in this period were mostly reported from a single outbreak from a religious group, which was not representative of the general population [14]. The major genomic type of the COVID-19 cases in this period was the S, V clade [15]. When the epidemiologic investigation was switched to a self-input questionnaire on 7 February 2022, the question asking whether the patient is an HCP was removed. Therefore, we included the cases until 4 February 2022, which was the end of the last full week collecting the relevant information.

In the comparison of country profiles, we reviewed cases, deaths, excess deaths, and vaccination rates from South Korea and several other countries, including Germany, Israel, Italy, Japan, the United Kingdom, and the United States, for comparison. Excess mortality is a parameter of assessing the impact of the pandemic to population health, since it serves as a comprehensive measure of the burden of death potentially related to COVID-19. Additionally, high excess death in a short period is associated with overstrained health services. We reviewed the P-score of excess death, which is comparable across countries with different sizes of population [7]. The selection criteria for the countries to review were based on practical considerations: Israel, the United Kingdom, and the United States were the countries with the earliest vaccination roll-out and loosening of non-pharmaceutical interventions (NPIs); Germany and the United Kingdom are countries in which the healthcare system is mainly public; Italy was one of the European countries reporting a large number of COVID-19 deaths at the beginning of the pandemic but with a remarkable recovery; and Japan is geographically close to Korea, and the detection of variants of concern (VOCs) from foreigners was always earlier in Japan than in Korea. 

### 2.3. Statistical Analysis

To compare the time trend of cases among HCP and the general population, we conducted a time series analysis. The time series analysis of COVID-19 cases and an ADF (Augmented Dickey–Fuller) test were performed using Python (version 3.10). Sex, age, and vaccination status in each group of HCP were compared and statistically evaluated via the Chi-square test using R (version 4.2.1).

## 3. Result

### 3.1. COVID-19 Cases and Deaths among HCP in Korea

COVID-19 cases among HCP in Korea accounted for 10,670 persons from 21 March 2020 to 4 February 2022, and 15 HCP died in the same period from COVID-19 (Table 1). The case fatality ratio was 0.14 (per 100) among HCP (Table 1), which was lower than the death rate among the total population of 0.75 (per 100) (shown in Table 2). Most cases were among nurses (*n* = 5898; 55.3%), followed by workers of the ‘others’ professional category (*n* = 3077; 28.8%) and doctors (*n* = 1695; 15.9%). In contrast, deaths were mostly reported among doctors (*n* = 9; 60%), followed by others (*n* = 4; 26.7%) and nurses (*n* = 2; 13.3%). While case numbers gradually increased as the pandemic progressed—a 51.7% increase during the Omicron-dominant period compared to the Delta-dominant period—deaths numbered the same, with six deaths occurring in the period of both Delta and Omicron.

The distributions of sex, age, and vaccination history were statistically different by professional category (*p* < 0.01 in all variables) (Table S1). Cases among doctors were more frequently in individuals who were male, older, and reluctant to be vaccinated than other professional categories. Nurses were mostly women (93%) and younger than the other groups—about 80% of them were younger than 50 years old. For the ‘others’ group, 76% were women, and age was similarly distributed in the range from 20 to 69.

### 3.2. The COVID-19 Pandemic in Korea’s General Population

In South Korea, 925,975 cases and 6930 deaths were reported from 21 March 2020 to 4 February 2022 (Table 2). As the pandemic progressed, the daily numbers of cases and deaths increased. The growth in the number of deaths was especially prominent in the latest period: 9 daily deaths in the Delta period vs. 44 daily deaths in the Omicron period. The daily numbers of cases increased during the Delta period compared to the Alpha period (2.45-fold increase), but the daily number of deaths during the Delta period was the same as that during the Alpha period.

**Figure 1:**
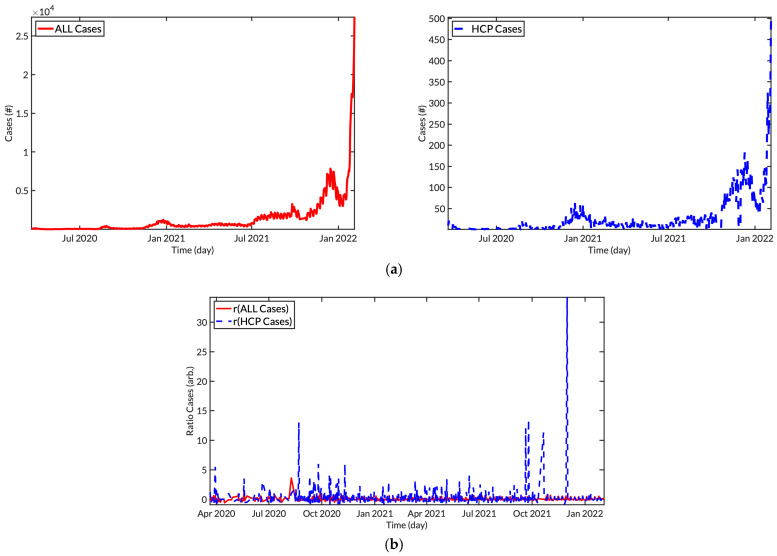
Daily trends of all COVID-19 cases (red line) and HCP COVID-19 cases (blue dotted line) (**a**), and the rate (**b**) reported in South Korea from 21 March 2020 to 4 February 2022.

We compared the daily trends of cases among all populations and among HCP (Figure 1). Not the distribution of absolute cases but the distribution of the rate of the daily cases was considered to make it stationary with the ADF test in the statistical model. The rate r was obtained via the difference in the number of cases between two days

$$r_{x}\equiv \frac{I_{x}\left(t\right)-I_{x}\left(t-1\right)}{I_{x}\left(t-1\right)},$$

in which x means either all or HCP cases, and t stands for time. Based on our assessment, the increase in HCP cases was followed 38 days after the increase in the general population (under the cross-correlation having a normalized maximum value of 0.189).

For two years, the pandemic situation has been modified by the effectiveness of nonpharmaceutical interventions and vaccination. Hence, we summarized the key events regarding the management of COVID-19 in Korea to create an understanding of the Korean situation by the provision of an overview (Table S2). Vaccination has rapidly increased since the rollout on 26 February 2021 in Korea, with a full vaccination rate of 78.8% achieved in about nine months. Quarantine and transmission management policy in the country was strengthened up to Dec 2020, and it has been repeatedly loosened and strengthened since then. Exemption from quarantine after entry into Korea for the fully vaccinated was in effect from 5 May 2021, and most NPIs in Korea were lifted from 1 November 2021, except for those limiting the number of people in a gathering to six (Seoul metropolitan area) or eight (other regions) and requiring a mask to be worn indoors and outdoors.

### 3.3. Indicators of the COVID-19 Pandemic in Selected Countries 

The total number of cases (per million) in Korea was the least (*n* = 3043) in June 2021, but it increased to 354,405 in June 2022, which was more than that in the U.S – the highest in 2021 (Table 3). However, the total number of deaths (per million) in Korea was the least in June 2021, and it maintained a lower level in June 2022, but Japan’s was the least. Excess mortality in Korea was relatively low both in 2021 and in 2022 compared to other countries. Moreover, Israel, Italy and Korea were the countries showing decreases in excess mortality between 2021 and 2022. Compared to six of the countries, South Korea was the last country to introduce vaccination, but its vaccination rate and numbers of vaccines administered were the highest among the seven countries.

### 3.4. Social, Economic, and Public Health Indicators of Selected Countries 

It is suggested that the situation of the pandemic in each country could be determined by the solidness of the infrastructure of the healthcare system in each country. Therefore, along with the profiles of COVID-19, we have also reviewed demographics and health-related variables in six countries (Table 4). The population in South Korea is about 51.8 million and the population density is 525.7, which is higher than those of the six comparison countries. The GDP per capita of Korea was USD 31.3K in 2020, ranking 10th in the world. Compared to that in 2019, real GDP growth in 2020 decreased by 0.9% in South Korea, which was the smallest loss among the compared countries. Objective indicators measuring health status—life expectancy, avoidable mortality, and obesity/obese rate—were close to the OECD average or better than the average. However, the proportion of people self-rating their health as ‘poor’ was the highest in Korea and worse than the OECD average. 

Access to care and the qualities of preventive care and primary care were average, but the quality of secondary care was worse than the OECD average. Health spending was in the range of the OECD average, although it was relatively low, but the number of hospital beds was higher than the average. The relative population of the number of people working in hospitals was the lowest in Korea. The total health workforce workers was at least twice as high in the United Kingdom and the United States compared to those in Israel, Italy, and Korea. In comparison with other countries, in Korea, the proportion of ‘nurses and midwives’ was the highest (range 30.1%–61.2%), and the proportion of ‘other staff’ was the lowest (range 2.4%–46.2%). In summary, while many profiles regarding the health system were better than or in the range of the OECD average, more Koreans were unsatisfied with their health, and the number of doctors per population was lower than the other countries.

## 4. Discussion

In Korea, the numbers of COVID-19 cases and deaths increased as the pandemic progressed. The leap in cases was large when Alpha became the dominant variant (4.53-fold compared to the previous GH clade period) and again when Omicron became dominant (5.14-fold compared to the previous Delta period). Alongside this trend in the general population, cases among HCP jumped after Alpha (4.11-fold compared to the GH clade period) and after Omicron (4.87-fold compared to the Delta period). The case fatality ratio decreased gradually in Korea, since the increase in deaths was not as large as the increase in cases. During the two years of the pandemic, the COVID-19 case fatality ratio (per 100) among HCP was lower than that among the general population in Korea, at 0.14 and 0.75, respectively.

In Korea, a strong COVID-19 monitoring system was applied to visitors to hospitals from February 2020 onward [16], such as obligations for all workers and visitors to hospitals to wear masks. Additionally, SARS-CoV-2 PCR testing was highly recommended for those with a history of exposure (visiting countries with reports of COVID-19 cases or meeting patients) or with any respiratory symptoms among HCP, which have contributed to early detection and to a lower infection rate among HCP compared to the general population.

In the United States, the case fatality ratio among HCP was 0.29 from 12 February 2020 to 9 April 2020 [1] and 0.95 from 12 February 2020 to 16 July 2020 [17], which were higher than our results. The data completeness of answering/checking whether cases were occurring in HCP or not varied by jurisdiction in the U.S. from 11% to 70% [17]. Therefore, if the under-reporting of HCP status is considered, the real case fatality ratio among HCP in the U.S. would be less than that reported. In fact, COVID-19 deaths among HCP in the U.S. declined after April 2020 and have remained flat since then [18]. In a report from Germany with 12,393 cases of SARS-CoV-2 infection until 25 May 2020 [19], the case fatality ratio among all HCP was 0.2% and was 0.5% among HCP who work at healthcare facilities such as nursing homes.

In previous studies, HCP at a higher risk of death were older, which is coherent with our study. Other variables related to a higher risk of death among HCP in the U.S. are being male, being of Black or Asian ethnicity, and having an underlying medical condition [17,18]. The occupational setting with the highest infection rate in our study was nurses and nurse assistants, which is consistent with the trend in the U.S. [17]. In the first community outbreak of SARS-CoV-2 in Daegu, the third-largest city in South Korea, a total of 121 doctors, nurses, and nurse assistants were infected with SARS-CoV-2, while 6620 cases were detected in the city from 7 February 2020 to 24 March 2020; thus, the infection rates were 4.4 per 1000 among HCP and 2.7 per 1000 in the general population [20]. The rate was lower in doctors (2.4 per 1000) and higher in nurses and nurse assistants (5.0 per 1000), suggesting that those in jobs involving more frequent encounters with patients had a higher risk of infection, which is coherent with our study result. In our study, among 10,670 HCP cases, nurses and nurse assistants formed the group with the highest incidence, followed by the ‘others’ group, and the least infected group was doctors.

We included a diverse range of HCP occupations in our analysis, including technicians and therapists in hospitals, pharmacists, first responders, care workers, etc., who had a chance of occupational exposure to SARS-CoV-2 by encountering COVID-19 patients at hospitals or at workplaces for public health services. By applying a broad inclusion criterion, we aimed to evaluate the SARS-CoV-2 infection rate among healthcare providers by professional category in Korea. In particular, we included care workers since residents and workers at caring facilities are at a higher risk of infection and transmission [21]. From this point of view, in a previous report from Korea, all 26 cases of nosocomial infections among HCP in Daegu from 7 February 2020 to 24 March 2020 were reported from long-term care facilities [20]. 

Pandemic trends and management policies regarding COVID-19 varied by country. Japan and Korea remained low in excess mortality, possibly resulting from relatively strong NPIs. In contrast, in Germany and the United Kingdom, excess deaths were relatively high in 2022, and elevation between 2021 and 2022 was observed. Excess death in the United Kingdom from March 2020 to June 2022 was supposed to be driven mainly by COVID-19, since excess death remained above the five-year average when the underlying cause of COVID-19 was removed from the total [22]. Based on a recent report from Germany’s statistical office (Statistisches Bundesamt (Destatis)), increased mortality figures have been observed since April 2020 [23]. Actually, an increase in excess mortality was observed in many countries, and cumulative excess deaths worldwide was estimated as 14.9M (95% UI 13.3M–16.7M) as of 31 December 2021 from the WHO, which resulted from a consistent increase since early 2020 [7].

It is very difficult to identify the determinant factors of the pandemic to be controlled, but governance, social awareness, and public health infrastructure would be among the key systemic factors [24]. The aggressiveness of personal sanitary behaviors and social/familial cultures with regard to private gatherings are different by country or by society, which might have affected the transmission of SARS-CoV-2 differently in the community. In addition, vaccination is the major contributing factor preventing the transmission of SARS-CoV-2, so the vaccination rate and vaccine hesitancy are the main monitoring indicators for the management of the pandemic since the rollout of vaccines. Among the countries compared in this study, Korea did not have the earliest rollout of SARS-CoV-2 vaccines, and its full vaccination rate was the lowest at 10% in June 2021, but its vaccination participation rate increased to be the highest rate of 86% in June 2022. Considering the population in Korea, its increase in COVID-19 cases was the steepest, but its death rate was one of the lowest; meanwhile, its participation in vaccination increased rapidly and became the highest.

The distribution of the categories of health workforce was different in each country. Nurses and midwives represented the largest category of care providers in hospitals in most countries except Israel. The sum of ‘other healthcare providers’ and ‘other staff’ was larger than the ‘nurses and midwives’ in most countries except Korea. Meanwhile, many countries recruited additional staff during the COVID-19 pandemic, which were exposed to a higher risk of being infected. Therefore, strengthening protection and the improvement of the working environment for these groups should be considered along with care plans for doctors and nurses.

After the outbreak of MERS in 2015, Korea strengthened infection prevention and control systems in hospitals, including training HCP, expanding negative-pressure isolation rooms, and stocking up on personal protective equipment (PPE) in preparation for superspreading events in hospitals and to protect HCP [25]. During the 2-month outbreak of MERS, 186 cases and 38 fatalities were reported in Korea [26]. Most of the cases (93%, 173/186) were nosocomial infections [27], and 20.9% (39/186) of all cases were among HCP [26]. After the MERS outbreak in 2015, the KCDC—now the KDCA—reorganized the Infectious Disease Risk Alert System, reformed the strategy of counteracting emerging infectious disease, and expanded the infrastructure of governing and testing facilities and the hiring of epidemiologic investigators. 

Based on these lessons, the Korean government not only set up the emergency administrative governance as quickly as possible but also introduced a rapid and broad tracing, testing, and treatment strategy [15,28,29]. As the official notice from the WHO was conveyed on 31 December 2019, soon after the Country Office in the People’s Republic of China reported cases of “pneumonia of unknown cause” to the IHR of WPRO [30,31], the Korean government officially announced Level 1 (attention: strengthen monitoring) of the Infectious Disease Risk Alert System on 3 January 2020, and countermeasure teams at the KCDC were formed for the surveillance, monitoring, and quarantine of SARS-CoV-2 cases. As the first patient was diagnosed in Korea on 20 January 2020, the alert level increased to Level 2 (caution: management of outbreak), and a Central Disease Control Headquarters was formed in the KCDC with the aim of strengthening surveillance and expanding testing infrastructure and epidemiologic investigations. COVID-19 cases increased to four people in Seoul in about a week, and the government increased the alert level to Level 3 (alertness: management of community outbreak) on 27 January 2020. The Central Accident Response Headquarters, serving at the direction of the Minister of Health and Welfare, was formed to prepare for the nationwide management of the outbreak. As cases surged from a religious group named Sincheonji in Daegu from 20 February 2020, Level 4, the highest level (seriousness: management of national spread) of the Infectious Disease Risk Alert System, came into effect from 23 February 2020. Accordingly, the Central Disaster and Safety Countermeasure Headquarters, serving at the direction of the Prime Minister, was formed as a comprehensive counteractor throughout the country, cooperating with the local governments and ministries. It was the first use of the Level 4 alarm in eleven years since the 2009 H1N1 pandemic in Korea. While strengthening NPIs, including quarantine policy, was effective in delaying the entrance of the variant [32], the pandemic progressed from local outbreaks to the whole country.

Along with the increase in the number of SARS-CoV-2 cases, the physical and psychological health of HCP caring for COVID-19 patients deteriorated [33,34,35]. From an in-depth interview of eighteen nurses caring for COVID-19 patients in designated hospitals for infectious diseases in Korea, extensive work and less appreciation reduced their motivation to work, while moral support from families, peers, and the public was an encouraging factor for keeping up the work [36]. Most of the difficulties felt in response to the COVID-19 outbreak related to a shortage of trained HCP in emerging infectious disease: the lack of an attending physician (64%), lack of ICPs (44%), lack of PPE (41%), and lack of AIIRs (32%) [37]. In previous studies assessing the knowledge, attitude, and practice of COVID-19, training on PPE use was associated with a better practice and with a positive attitude among healthcare workers, which is necessary in response to COVID-19 infection at hospitals [38,39].

In this paper, we aimed to describe the burden of HCP in Korea by analyzing national data. Nevertheless, unless the HCP were diagnosed at their workplace, such as clinics, hospitals, or institutions of health services, it is possible that cases among HCP were undisclosed. For this reason, we cannot exclude the possibility of the under-reporting of COVID-19 cases among HCP in Korea. We speculate that any under-reporting bias, if present, would be differential. Doctors and nurses are professionals holding a license from the government and are considered officially as HCP, so these workers will answer affirmatively when asked if they are HCP or not. In contrast, for men and women working in professions of the ‘others’ group, even though they work for health services and hold a certificate of qualification appropriate to their work, some of them may not feel obligated to identify themselves as HCP. Furthermore, some care workers who assist patients in wards and during overnight stays in hospitals are not necessarily asked to acquire certificates, nor do they necessarily work regularly, so some may have answered as working at health facilities, but some may not have.

The other limitation of our study is that we could not analyze epidemiologic variables such as the transmission route, severity of disease, and risk factors of death with the long-term data. The route of transmission was collected in our data, but missing or unclassified cases increased to comprise over 30% of all cases as case numbers surged. In addition, we limited the numbers of countries for comparison, and a narrative review was conducted. Therefore, a qualitative summary rather than a comprehensive systematic review was provided.

It is supposed that the route of infection among HCP derived mainly from the community as the size of pandemic grew. Nevertheless, HCP are still at a higher risk of infection because of the greater possibility of direct contact with patients at work, compared to those who are not at risk occupationally. We suggest results from long-term data of COVID-19 among health workers may add knowledge to the area of health worker protection when coping with an outbreak of a respiratory infectious disease.

## Figures and Tables

**Table 1:** COVID-19 cases and deaths among healthcare professionals in Korea from 21 March 2020 to 4 February 2022.

Variables		Period by the Major Variant of SARS-CoV-2								Total	
		21 March 2020~12 December 2020		13 December 2020~7 April 2021		8 April 2021~24 November 2021		25 November 2021~4 February 2022		21 March 2020~4 February 2022	
		(GH Clade)		(Alpha)		(Delta)		(Omicron)		-	
		N	%	N	%	N	%	N	%	N	%
Cases by professional category											
	Total	749	100	1339	100	3409	100	5173	100	10,670	100
	Doctors ^1^	113	15.1	210	15.7	550	16.1	822	15.9	1695	15.9
	Nurses ^2^	427	57.0	734	54.8	1881	55.2	2856	55.2	5898	55.3
	Others ^3^	209	27.9	395	29.5	978	28.6	1495	28.9	3077	28.8
Deaths by professional category											
	Total	1	100	2	100	6	100	6	100	15	100
	Doctors ^1^	1	100	1	50.0	4	67.0	3	50.0	9	60.0
	Nurses ^2^	0	-	1	50.0	1	16.5	0	-	2	13.3
	Others ^3^	0	-	0	-	1	16.5	3	50.0	4	26.7
Deaths over cases per 100 ^4^		0.13		0.15		0.18		0.12		0.14	

^1^ Medical doctors, dentists, and oriental doctors. ^2^ Registered nurses and nurse assistants. ^3^ Physical therapists, radiologic technicians, emergency medical technicians, medical laboratory technologists, occupational therapists, psychologists, dental assistants, care workers, pharmacists, herbalists, paramedics, and firefighters. ^4^ Deaths divided by cases (per 100).

**Table 2:** COVID-19 cases and deaths in Korea by periods of major variants from 21 March 2020 to 4 February 2022.

Variables		Period by Major Variant of SARS-CoV-2				Total
		21 March 2020~12 December 2020	13 December 2020~7 April 2021	8 April 2021~24 November 2021	25 November 2021~4 February 2022	21 March 2020~4 February 2022
		(GH Clade)	(Alpha)	(Delta)	(Omicron)	-
Cases ^1^						
	Total cases	33,076	65,163	318,164	509,572	925,975
	Daily cases	124	562	1377	7077	1350
Deaths ^1^						
	Total deaths	592	1029	2156	3153	6930
	Daily deaths	2	9	9	44	10
Deaths over cases per 100 ^2^		1.79	1.58	0.68	0.62	0.75

^1^ Extracted from OurWorldInData.org [7]. ^2^ Total deaths divided by total cases (per 100).

**Table 3:** Profiles of the COVID-19 pandemic in selected countries.

Dimension/Indicator	Germany	Israel	Italy	Japan	South Korea	United Kingdom	United States
Total COVID-19 cases (per million) ^1^							
30 June 2021	44,697	90,601	71,908	6416	3043	71,420	100,226
30 June 2022	339,221	468,521	312,677	74,767	354,405	338,877	260,171
Total COVID-19 deaths (per million) ^1^							
30 June 2021	1089	691	2153	118	38	1905	1782
30 June 2022	1693	1179	2842	251	474	2683	3019
Excess mortality due to all causes (P-score) ^2^							
27 June 2021	1.58	8.89	15.36	2.56	2.17	−6.68	5.76
26 June 2022	11.44	3.69	11.69	3.53	−0.61	13.77	7.01
Vaccination rollout date	27 December 2020	20 December 2020	27 December 2020	17 February 2021	26 February 2021	8 December 2020	14 December 2020
Full vaccination rate (%) ^1^							
30 June 2021	37.52	55.96	32.01	14.71	10.03	49.12	49.14
30 June 2022	75.95	66.15	80.93	82.15	86.11	74.6	67.03
Total vaccinations per hundred ^1^							
30 June 2021	91.98	116.58	88.05	42.76	37.66	115.8	102.04
30 June 2022	219.25	195.78	233.48	228.66	243.08	222.59	180.06
Total boosters per hundred ^1^							
30 June 2021	0.01	-	-	-	-	-	-
30 June 2022	68.47	57.38	69.33	63.25	72.93	59.45	37.44

^1^ Extracted from OurWorldInData.org [10]. ^2^ The P-score is the percentage difference between the reported number of weekly deaths and the projected number of deaths for the same period based on previous years, except for Japan, for which we have the monthly data for June 30 in 2021 or in 2022 [7].

**Table 4:** Socio-economic factors and healthcare profiles in selected countries.

Dimension/Indicator		Germany	Israel	Italy	Japan	South Korea	United Kingdom	United States
Demographics ^1^ (2020)								
	Population (million persons)	83.2	9.2	59.4	125.7	51.8	67.1	329.5
	Density of population	239.8	400	203.1	347	525.7	280.6	36.2
	Elderly population (≥65)	21.9	12.1	23.4	28.8	15.7	18.6	16.9
	Old-age dependency ratio	36.5	23.9	39.5	52	23.6	32	28.4
	Female proportion (%)	50.6	50.2	51.3	51.2	49.9	50.6	50.5
Economic status ^2^ (2020)								
	GDP per capita in USD	41.3K	37.5K	29.4K	34.8K	31.3K	43K	58.2K
	Real GDP growth rate (vs. 2019)	−4.6	−2.2	−8.9	−4.6	−0.9	−9.4	−3.4
Health status ^3^ (2019)								
	Life expectancy	81.4	82.9	83.6 ^B^	84.4 ^B^	83.3	81.4	78.9
	Avoidable mortality	175	125 ^B^	136 ^B^	130 ^B^	139 ^B^	188	265 ^W^
	Population in poor health (%)	8.5	11	7	13.6 ^W^	15.2 ^W^	7.4	3.3 ^B^
Risk factor ^3^ (2019)								
	Proportion of overweight/obesity	60	50.9	46.4	27.2 ^B^	33.7 ^B^	64.2	73.1 ^W^
Access to care ^3^ (2019)								
	Financial protection (Compulsory prepayment) (%)	84.6 ^B^	64.8	73.8	83.8 ^B^	61	78.5	82.7
Quality of care ^3^ (2019)								
	Effective primary care (Avoidable COPD admission)	250	155	39 ^B^	NR	152	223	194
	Effective preventive care (Mammography within 2 years) (%)	50.1	72.1	60.7	44.6 ^W^	70.2	75.1	76.5
	Effective secondary care (30-day mortality of AMI)	8.3	5.3	5.4	9.7 ^W^	8.9 ^W^	6.6	4.9
Health system capacity and resources ^3^ (2019)								
	Health spending (per capita in USD)	6518 ^B^	2903	3653	4691	3406	4500	10,948 ^B^
	Hospital beds (per 1000 population)	7.9 ^B^	3	3.2	12.8 ^B^	12.4 ^B^	2.5	2.8 ^W^
	Doctors (per 1000 population)	4.4	3.3	4.1	2.5 ^W^	2.5 ^W^	3	2.6
	Nurses (per 1000 population)	13.9 ^B^	5	6.2	11.8	7.9	8.2	12
Health workforce (per 1000) ^1,4^ (2019)								
	Physicians	2.5	2.1	2.3	1.7	1.2	2.2	1.0
	Nurses and midwives	6.9	3.1	4.5	7.5	5.2	7.6	8.3
	Healthcare assistants	-	0.2	-	1.4	0	1.4	-
	Other health service providers	3.9	1.6	1.3	3.6	1.8	7.7	2.0
	Other staff	3.8	3.4	2.7	2.3	0.2	4.2	9.8
	Total	17.1	10.3	10.7	16.5	8.5	23.1	21.2

^1^ Extracted from OECD Health statistics [8]. ^2^ Extracted from OurWorldInData.org [7]. ^3^ Extracted from OECD Health at a Glance 2021 [9]. ‘NR’, not reported; uppercase ‘B’, better than OECD average; uppercase ‘W’, worse than OECD average, unless close to OECD average. ^4^ Permanent location of this file: https://stat.link/sr4y1w (accessed on 1 June 2023). The fact that Japan’s data refer to full-time equivalent rather than head count possibly resulted in an underestimation.

## Data Availability

Data can be made available upon request.

## Supplementary Materials

The following supporting information can be downloaded at: https://www.mdpi.com/article/10.3390/tropicalmed8060308/s1, Table S1. Characteristics of COVID-19 cases among healthcare professionals in Korea from 21 March 2020 to 4 February 2022; Table S2. Summary of the key characteristics of the COVID-19 epidemic in Korea by periods of major variants from 21 March 2020 to 4 February 2022.

## References

[1] CDC COVID-19 Response Team (2020). Characteristics of Health Care Personnel with COVID-19—United States, February 12–April 9, 2020. Morb. Mortal. Wkly. Rep..

[2] Iversen K., Bundgaard H., Hasselbalch R.B., Kristensen J.H., Nielsen P.B., Pries-Heje M., Knudsen A.D., E Christensen C., Fogh K., Norsk J.B. (2020). Risk of COVID-19 in health-care workers in Denmark: An observational cohort study. Lancet Infect. Dis..

[3] Reusken C.B., Buiting A., Bleeker-Rovers C., Diederen B., Hooiveld M., Friesema I., Koopmans M., Kortbeek T., Lutgens S.P., Meijer A. (2020). Rapid assessment of regional SARS-CoV-2 community transmission through a convenience sample of healthcare workers, the Netherlands, March 2020. Eurosurveillance.

[4] Billings J., Ching B.C.F., Gkofa V., Greene T., Bloomfield M. (2021). Experiences of frontline healthcare workers and their views about support during COVID-19 and previous pandemics: A systematic review and qualitative meta-synthesis. BMC Health Serv. Res..

[5] Chemali S., Mari-Sáez A., El Bcheraoui C., Weishaar H. (2022). Health care workers’ experiences during the COVID-19 pandemic: A scoping review. Hum. Resour. Health.

[6] Gómez-Ochoa S.A., Franco O.H., Rojas L.Z., Raguindin P.F., Roa-Díaz Z.M., Wyssmann B.M., Guevara S.L.R., Echeverría L.E., Glisic M., Muka T. (2021). COVID-19 in Health-Care Workers: A Living Systematic Review and Me-ta-Analysis of Prevalence, Risk Factors, Clinical Characteristics, and Outcomes. Am. J. Epidemiol..

[7] Excess Mortality during the Coronavirus Pandemic (COVID-19) 2020. https://ourworldindata.org/.

[8] OECD (2021). Health at a Glance 2021.

[9] OECD Health Statistics (2022). https://www.oecd.org/els/health-systems/health-data.htm.

[10] (2022). Coronavirus Pandemic (COVID-19) [Database Online]: OurWorldInData.org. https://ourworldindata.org/coronavirus.

[11] Volz E., Mishra S., Chand M., Barrett J.C., Johnson R., Geidelberg L., Hinsley W.R., Laydon D.J., Dabrera G., O’Toole Á. (2021). Assessing transmissibility of SARS-CoV-2 lineage B.1.1.7 in England. Nature.

[12] Davis-Gardner M.E., Lai L., Wali B., Samaha H., Solis D., Lee M., Porter-Morrison A., Hentenaar I.T., Yamamoto F., Godbole S. (2023). Neutralization against BA.2.75.2, BQ.1.1, and XBB from mRNA Bivalent Booster. N. Engl. J. Med..

[13] Park A.K., Kim I.H., Kim J., Kim J.M., Kim H.M., Lee C.Y., Han M.G., Rhie G.E., Kwon D., Nam J.G. (2021). Genomic Surveillance of SARS-CoV-2: Distribution of Clades in the Republic of Korea in 2020. Osong Public Health Res. Perspect..

[14] (2020). Korean Society of Infectious Diseases, Korean Society of Pediatric Infectious Diseases, Korean Society of Epidemiology, Korean Society of Antimicrobial Therapy, Korean Society for Healthcare-associated Infection Control and Prevention, Korean Centers for Disease Control and Prevention. Report on the Epidemiological Features of Coronavirus Disease 2019 (COVID-19) Outbreak in the Republic of Korea from January 19 to March 2, 2020. J. Korean Med. Sci..

[15] Kim I.H., Park A.K., Kim J.M., Kim H.M., Lee N.J., Woo S.H., Lee C.Y., Lee J.H., Rhee J.E., Kim E.J. (2021). COVID-19 variant surveillance study in the Republic of Korea. Public Health Wkly. Rep..

[16] Jeon Y.W., Park E.S., Jung S.J., Kim Y., Choi J.Y., Kim H.C. (2020). Protection of Healthcare Workers Against COVID-19 at a Large Teaching Hospital in Seoul, Korea. Yonsei Med. J..

[17] Hughes M.M., Groenewold M.R., Lessem S.E., Xu K., Ussery E.N., Wiegand R.E., Qin X., Do T., Thomas D., Tsai S. (2020). Update: Characteristics of Health Care Personnel with COVID-19—United States, February 12–July 16, 2020. Morb. Mortal. Wkly. Rep..

[18] Lin S., Deng X., Ryan I., Zhang K., Zhang W., Oghaghare E., Gayle D.B., Shaw B. (2022). COVID-19 Symptoms and Deaths among Healthcare Workers, United States. Emerg. Infect. Dis..

[19] Nienhaus A., Hod R. (2020). COVID-19 among Health Workers in Germany and Malaysia. Int. J. Environ. Res. Public Health.

[20] Kim J.-H., An J.A.-R., Min P.-K., Bitton A., Gawande A.A. (2020). How South Korea Responded to the COVID-19 Outbreak in Daegu. NEJM Catal Innov Care Deliv..

[21] Thompson D.-C., Barbu M.-G., Beiu C., Popa L.G., Mihai M.M., Berteanu M., Popescu M.N. (2020). The Impact of COVID-19 Pandemic on Long-Term Care Facilities Worldwide: An Overview on International Issues. BioMed Res. Int..

[22] (2022). Excess deaths in England and Wales: March 2020 to June 2022. Statistical Bulletin.

[23] Number of Deaths and Excess Mortality (2023). Destatis Website: Statistisches Bundesamt (Destatis). https://www.destatis.de/EN/Themes/Cross-Section/Corona/Society/population_death.html.

[24] Tsou H.-H., Kuo S.-C., Lin Y.-H., Hsiung C.A., Chiou H.-Y., Chen W.J., Wu S.-I., Sytwu H.-K., Chen P.-C., Wu M.-H. (2022). A comprehensive evaluation of COVID-19 policies and outcomes in 50 countries and territories. Sci. Rep..

[25] Jeon Y., Kim Y. (2022). COVID-19: Protecting Health-Care Workers in South Korea. Disaster Med. Public Health Prep..

[26] Oh M.-D., Park W.B., Park S.-W., Choe P.G., Bang J.H., Song K.-H., Kim E.S., BIN Kim H., Kim N.J. (2018). Middle East respiratory syndrome: What we learned from the 2015 outbreak in the Republic of Korea. Korean J. Intern. Med..

[27] Yang T.U., Noh J.Y., Song J.-Y., Cheong H.J., Kim W.J. (2021). How lessons learned from the 2015 Middle East respiratory syndrome out-break affected the response to coronavirus disease 2019 in the Republic of Korea FAU—Yang, Tae Un FAU—Noh, Ji Yun FAU—Song, Joon-Young FAU—Cheong, Hee Jin FAU—Kim, Woo Joo. Korean J. Intern. Med..

[28] Kim I., Lee J., Lee J., Shin E., Chu C., Lee S.K. (2020). KCDC Risk Assessments on the Initial Phase of the COVID-19 Outbreak in Korea. Osong Public Health Res. Perspect..

[29] Kim T. (2020). Improving Preparedness for and Response to Coronavirus Disease 19 (COVID-19) in Long-Term Care Hospitals in Korea. Infect. Chemother..

[30] WHO (2020). Listings of WHO’s Response to COVID-19. https://www.who.int/news/item/29-06-2020-covidtimeline.

[31] Worobey M. (2021). Dissecting the early COVID-19 cases in Wuhan. Science.

[32] Kwon J.H., Kim J.M., Lee D.H., Park A.K., Kim I.H., Kim D.W., Kim J.Y., Lim N., Cho K.Y., Kim H.M. (2021). Genomic epidemiology reveals the reduction of the introduction and spread of SARS-CoV-2 after implementing control strategies in Republic of Korea, 2020. Virus Evol..

[33] Robles R., Rodríguez E., Vega-Ramírez H., Álvarez-Icaza D., Madrigal E., Durand S., Morales-Chainé S., Astudillo C., Real-Ramírez J., Medina-Mora M.-E. (2021). Mental health problems among healthcare workers involved with the COVID-19 outbreak. Rev. Bras. de Psiquiatr..

[34] Rodríguez B.O., Sánchez T.L. (2020). The Psychosocial Impact of COVID-19 on health care workers. Int. Braz. J. Urol..

[35] Søvold L.E., Naslund J.A., Kousoulis A.A., Saxena S., Qoronfleh M.W., Grobler C., Münter L. (2021). Prioritizing the Mental Health and Well-Being of Healthcare Workers: An Urgent Global Public Health Priority. Front. Public Health.

[36] Lee N., Lee H.-J. (2020). South Korean Nurses’ Experiences with Patient Care at a COVID-19-Designated Hospital: Growth after the Frontline Battle against an Infectious Disease Pandemic. Int. J. Environ. Res. Public Health.

[37] Park S.Y., Kim B., Jung D.S., Jung S.I., Oh W.S., Kim S.W., Peck K.R., Chang H.H., Korean Society of Infectious Diseases (2020). Psychological distress among infectious disease physicians during the response to the COVID-19 outbreak in the Republic of Korea. BMC Public Health.

[38] Limbu D.K., Piryani R.M., Sunny A.K. (2020). Healthcare workers’ knowledge, attitude and practices during the COVID-19 pandemic response in a tertiary care hospital of Nepal. PLoS ONE.

[39] Rainford L., Zanardo M., Buissink C., Decoster R., Hennessy W., Knapp K., Kraus B., Lanca L., Lewis S., Mahlaola T. (2020). The impact of COVID-19 upon student radiographers and clinical training. Radiography.

