# Supplementary material for: COVID-19 Cases and Deaths among Healthcare Personnel with the Progression of the Pandemic in Korea from March 2020 to February 2022
Source: Trop Med Infect Dis. 2023 Jun 5;8(6):308. doi: 10.3390/tropicalmed8060308 (PMC10302575; doi:10.3390/tropicalmed8060308)
Supplement: Supplementary file 1 [file tropicalmed-08-00308-s001.zip › tropicalmed-2398999-supplementary.pdf]

Table S1. Characteristics of COVID-19 cases among healthcare professionals in Korea from 21 March 2020 to 4 February 2022.

**Variables**	**Doctors [1]**		**Nurses [2]**		**Others [3]**		***p*-Value**
	**N**	**%**	**N**	**%**	**N**	**%**	
Sex							<0.01
Male	1238	73.0	415	7.0	739	24.0	
Female	457	27.0	5483	93.0	2338	76.0	
Age							<0.01
<20	0	0.0	22	0.4	0	0.0	
20–29	164	9.7	1841	31.2	809	26.3	
30–39	523	30.9	1377	23.3	563	18.3	
40–49	458	27.0	1426	24.2	357	11.6	
50–59	324	19.1	989	16.8	546	17.7	
60–69	162	9.6	225	3.8	723	23.5	
70+	64	3.8	18	0.3	79	2.6	
Vaccination at diagnosis							<0.01
Partially vaccinated	130	7.7	337	5.7	166	5.4	
Fully vaccinated	994	58.6	3658	62.0	1928	62.7	
Booster vaccinated	429	25.3	1670	28.3	805	26.2	
Not vaccinated	142	8.4	233	4.0	178	5.8	

[1] Medical doctors, dentists, and oriental doctors. [2] Registered nurses and nurse assistants. [3] Physical therapists, radiologic technicians, emergency medical technicians, medical laboratory technologists, occupational therapists, psychologists, dental assistants, pharmacists, care workers, licensed herbalists, paramedics, and firefighters.

Table S2. Summary of the key characteristics of the COVID-19 epidemic in Korea by periods of major variants from March 21, 2020 to February 4, 2022.

Variables	Period by major variant of SARS-CoV-2			
	20.3.21~ 20.12.12 (GH clade)	20.12.13~ 21.4.7 (Alpha)	21.4.8~ 21.11.24 (Delta)	21.11.25~ 22.2.4 (Omicron)
Main policies				
Major immigration and quarantine policy of Incheon airport [1]	Quarantine of 14 days required upon arrival from April 1.<br><br>Monitoring of foreign countries in three groups established: high risk, moderate, and safe.<br><br>Designation of four high-risk countries effective from Jul 13. This was expanded to six countries from Jul 20.	The category 'closely monitoring countries' was added to the monitoring system of the pandemic in foreign countries from Oct 2020.	Fully vaccinated exempted from quarantine from May 5 (limited to those vaccinated in Korea), and from Jul 1 (expanded to those vaccinated outside Korea).<br><br>Quarantine of 14 days reduced to 10 days from Nov 1.<br><br>Foreign country monitoring categories modified from four to three groups from Nov 1.	Passengers arriving from 8 countries in Africa reporting Omicron not allowed to enter from Nov 28, expanded to 11 countries on Dec 17. This was lifted on Feb 4, 2022.<br><br>Quarantine days reduced to 7 days from Feb 4 and later lifted on Jun 8, 2022.
Social distancing policy in general [2]	Mobility restraints in Daegu city—where an outbreak occurred—for two weeks from Feb 22.<br><br>Schools closed from March, with remote working and prohibition of gatherings in effect from March 22 until May 5. Later, NPI was downgraded but public places were closed.	Loosened to level 2.0 in Feb (level 1.5 in most municipalities outside metropolitan areas).	Strengthened to the highest level in mid-Jul, which included the closure of public facilities and prohibition of mass gatherings.<br><br>From Nov 1, most NPI started to be lifted, except for mask requirements and limiting the number of people in private meetings.	From Dec 18, micro-modified to a more restrictive level.<br><br>From Apr 25, every NPI was lifted, except the wearing of masks indoors.

[1] This table conveys a short description of the overall policy. The guidelines consisted of detailed information, including exception criteria in several rules, but these are not described in the table.

[2] A general guideline for continuing or adjusting the level of NPI was used in all cities/municipalities in Korea. Since December 2020, local governments have been able to modify the NPI level in their city/municipality.
